# Testing Commercial Sex Workers for Sexually Transmitted Infections in Victoria, Australia: An Evaluation of the Impact of Reducing the Frequency of Testing

**DOI:** 10.1371/journal.pone.0103081

**Published:** 2014-07-21

**Authors:** Eric P. F. Chow, Glenda Fehler, Marcus Y. Chen, Catriona S. Bradshaw, Ian Denham, Matthew G. Law, Christopher K. Fairley

**Affiliations:** 1 Melbourne Sexual Health Centre, Alfred Health, Carlton, Australia; 2 Central Clinical School, Faculty of Medicine, Nursing and Health Sciences, Monash University, Melbourne, Australia; 3 The Kirby Institute, University of New South Wales, Sydney, Australia; State University of Maringá/Universidade Estadual de Maringá, Brazil

## Abstract

**Background:**

The frequency of testing sex workers for sexually transmitted infections (STIs) in Victoria, Australia, was changed from monthly to quarterly on 6 October 2012. Our aim was to determine the impact of this change to the clients seen at the Melbourne Sexual Health Centre (MHSC).

**Methods:**

Computerised medical records of all clients attending at MHSC from 7 October 2011 to 7 October 2013 were analysed.

**Results:**

Comparing between the monthly and quarterly testing periods, the number of consultations at MSHC with female sex workers (FSW) halved from 6146 to 3453 (*p*<0.001) and the consultation time spent on FSW reduced by 40.6% (1942 h to 1153 h). More heterosexual men (*p*<0.001), and women (*p*<0.001) were seen in the quarterly testing period. The number of STIs diagnosed in the clinic increased from 2243 to 2589 from the monthly to quarterly period, respectively [15.4% increase (*p*<0.001)]. Up to AU$247,000 was saved on FSW testing after the shift to quarterly testing.

**Conclusions:**

The change to STIs screening frequency for sex workers from monthly to quarterly resulted in a 15% increase in STI diagnoses in the clinic and approximate a quarter of a million dollars was diverted from FSW testing to other clients. Overall the change in frequency is likely to have had a beneficial effect on STI control in Victoria.

## Introduction

Laws governing the sex industry in Australia are determined by the State and Territory Governments and vary across the country. In Victoria, under the Sex Work Act 1994 and Sex Work Regulations 2006, it is an offence for a sex worker to work if they know they have HIV or one of the other sexually transmitted infections (STI) prescribed in the Sex Work Regulations 2006. By law, a sex worker is presumed to know that they had an STI unless, they had been having regular HIV and STI testing and they believed on ‘reasonable grounds’ that they were not infected [1]. Prior to 6 October 2012, monthly STI screening for *Chlamydia trachomatis*, *Neisseria gonorrhoea*, and *Trichomonas vaginalis* and quarterly serological tests for HIV and syphilis was recommended for Victorian sex workers. From October 2012, the recommended frequency of STI screening for sex workers changed from monthly to every three months (quarterly). At the time of screening, sex workers are issued with a certificate of attendance for STI screening which they can present to the brothel manager. In some brothels, they are not permitted to work without an in-date certificate of screening.

Sex workers in Victoria have an extremely low STI prevalence. Among 4296 sex workers who attended at the Melbourne Sexual Health Centre (MSHC) for the first time between 2002 and 2011, 0.02% had HIV, 0.06% had syphilis, 0.3% had gonorrhoea and 3.7% had chlamydia diagnosed [2], which is lower than the estimated rate in the general community [3], [4]. Sex workers have an extremely high rate of condom use (nearly 100%) with their commercial clients, suggesting sex workers mainly acquire STI from their regular non-commercial partners instead of from their commercial clients [5].

STI screening of sex workers can ensure earlier detection of STIs and play an important role in STI control. However, the frequency of STI screening should depend on the local HIV/STI epidemic and nature of sex work [6]. Although frequent STI screening is an effective HIV/STI prevention program in some countries [6], [7], previous studies showed that the monthly STI screening of sex workers in low-prevalence setting, such as in Victoria, is not a cost effective strategy, and a waste of resources [8], [9]. In one study it was estimated that it costs AU$90,000 for each chlamydia case averted and each quality adjusted life year saved costs AU$10,000,000, well above the AU$50,000 threshold for good value [8]. It is hypothesized that reducing the frequency of STI screening of sex workers would result in more consultation time available for higher-risk individuals, such as men who have sex with men (MSM), and an overall improvement in population health as a result [9].

Due to the low STI incidence rate, the high rate of condom use in female sex workers (FSW) and the potential waste of limited public health resources; the Victorian Minister for Health published in the gazette an order to change to STI screening frequency in relation to sex workers from monthly to quarterly. To evaluate the impact the change in the screening regimen of sex workers has had in Victoria, we compared the number of STI diagnoses, nature and duration of clinical consultations seen at the MHSC in the year before and after the change. The aim of this study was to determine if the change in screening frequency of sex workers would lead to more high risk clients being seen, and more STIs being diagnosed at the main public sexual health clinic in Victoria.

## Methods

We conducted a retrospective study investigating all individuals attending the MSHC between 7 October 2011 and 7 October 2013. MSHC is the major public sexual health clinic and located in the city of Melbourne, Victoria, Australia. The clinic provides about 35,000 consultations annually. MSHC provides a walk-in service and all clients are triaged-in by a registered nurse. Clients who are at higher risk of infections or who have noticeable symptoms are prioritised to the service [10]. No referrals are required and all services are free-of-charge.

The demographic characteristics, epidemiological and sexual behavioural data, and clinical diagnoses of each client were recorded in the electronic medical system, the Clinic Practice Management System (CPMS), at each clinic visit.

Data were stratified into ‘monthly’ (from 7 October 2011 to 6 October 2012) and ‘quarterly’ (from 7 October 2012 to 7 October 2013) testing period. Clients were categorised into different risk groups according to their self-reported behaviours. Female sex workers (FSW) were defined as women who sell sex to men and have ever attended MSHC for a sex work certificate. A heterosexual male was considered as a man who has not had sex with a man since their first attendance at MSHC and has never attending for a sex work certificate; while a heterosexual female was defined as non-sex-working woman who has had male partners. Men who have sex with men (MSM) were defined as men who had sex with other men in the last 12 months or in the 12 months before any previous visit to MSHC. Men who have attended MSHC for a sex worker certificate or ever sold sex were defined as male sex workers. In addition, MSM who occasionally receive money for sex were also considered as male sex workers [11]. Clients with symptoms or who required STI treatments were seen by doctors only, and they were classified as ‘symptomatic clients’ for the sake of this study. However, clients without any symptoms were seen by doctors or nurses and this depends on the availability of the staff at the clinic.

Specific parameters in each risk group, including the number of clinical consultations, the time spent in clinical consultations, and the number of diagnoses of specific STI (chlamydia, gonorrhoea, *Mycoplasma genitalium*, syphilis, trichomonas, and HIV), were compared between the monthly and quarterly testing periods. The time spent in consultations for each client was measured from the start to the end of the each consultation and was collected automatically in CPMS. The rates of STI detection per 100 clinical consultations, and per 100 hours of consultation time in each risk group were calculated. Chi-square test or Fisher’s exact test was used to investigate the differences of the proportion in each risk categories of clients between the monthly and quarterly testing period. The 95% confidence intervals (CI) of the STI detection rates were calculated based on the ‘exact’ binomial distribution [12]. All statistical analyses were performed using Statistical Package for the Social Sciences (SPSS) software (version 20.0, SPSS Inc., Chicago, IL).

The total costs of FSW consultations and the laboratory tests were compared during the period. The costs of pathology services and clinical consultations were extracted from the Australian Medicare Health System [13]. Costs for each clinical consultation depend on the length of consultation. Consultations less than 20 mins were classified as level B and consultations more than 20 mins were level C consultations.

The study had ethical approval from the Alfred Hospital Ethics committee (number 433/13). No consent was given to the participants. The project involves previously collected clinical information and it is impracticable to obtain consent and the purpose of this project is to monitor and evaluate the health service. The original data is the client electronic epidemiological data and clinical record was collected during the clinical consultation and was collected for the clients' clinical care. Contacting more than 70,000 clients after they presented to ask their consent to review their clinical record would be impractical and may cause the client considerable concern or risk breaching their confidentiality. The data were analysed and reported anonymously.

### Ethics Approval

Ethics approval for this study was obtained by the Alfred Hospital Ethics Committee (No.: 433/13).

## Results

There was a small decrease (−4.1%) in the total number of clinical consultations at MSHC from the monthly (N = 36,260) to the quarterly (N = 34,775) testing period but a minimal reduction in consultation time (−0.6%) from 10020 h to 9962 h (Table 1). Compared to the monthly testing interval, the number of consultations with FSW halved (from 6146 to 3453; *p*<0.001) and the consultation time spent on FSW reduced by 40.6% (from 1942 h to 1153 h; *p*<0.001) in the quarterly testing period. Significant increases in the number of consultations on both heterosexual females (from 7309 to 8571; *p*<0.001) and males (from 9508 to 9827; *p*<0.001) were observed. There was no significant change in the number of consultations for MSM (from 13100 to 12724; *p* = 0.20) but a significant rise in consultation time for MSM (from 3496 h to 3749 h; *p*<0.001).

**Table 1:** Comparison of the number of consultations and consultation hours spent among different risk categories of clients at the MSHC in the monthly and quarterly testing period.

Clients risk group	Number of consultations			Consultation hours spent		
	Monthly testingperiod N (%)	Quarterly testingperiod N (%)	P-value	Monthly testingperiod Hour (%)	Quarterly testingperiod Hour (%)	P-value
**All clients**	36260 (100%)	34775 (100%)	–	10020 (100%)	9962 (100%)	–
**Heterosexual females**	7309 (20.2%)	8571 (24.6%)	<0.001***	2017 (20.1%)	2409 (24.2%)	<0.001***
**Heterosexual males**	9508 (26.2%)	9827 (28.3%)	<0.001***	2508 (25.0%)	2593 (26.0%)	0.11
**Female sex workers**	6146 (16.9%)	3453 (9.9%)	<0.001***	1942 (19.4%)	1153 (11.6%)	<0.001***
**Men who have sex** **with men**	13100 (36.1%)	12724 (36.6%)	0.20	3496 (34.9%)	3749 (37.6%)	<0.001***
**Male sex workers**	711 (2.0%)	500 (1.7%)	<0.001***	201 (2.0%)	155 (1.6%)	0.016*

*p<0.05;

**p<0.01;

***p<0.001.

The proportion of overall consultations that were for symptomatic presentations rose significantly (*p*<0.001) from 27.1% in the monthly testing period to 31.7% in the quarterly testing period (Table 2). The proportion of clinics consultations that were symptomatic MSM (from 7.8% to 9.3%; *p*<0.001), symptomatic heterosexual females (from 7.3% to 8.9%; p<0.001), and symptomatic heterosexual males (from 9.3% to 9.9%; *p*<0.001) increased significantly. In addition, more consultation time was spent on symptomatic heterosexual females (p<0.001), and MSM (p<0.001) in the quarterly testing period.

**Table 2:** Comparison of the number of consultations with symptomatic conditions among different risk categories of clients at the MSHC in the monthly and quarterly testing period.

Clients risk group	Number of consultations with symptomatic conditions			Consultation hours spent on symptomatic conditions		
	Monthly testingperiod (N = 36260)N (%)	Quarterly testingperiod (N = 34775)N (%)	P-value	Monthly testingperiod (10020 h)Hour (%)	Quarterly testingperiod (9962 h)Hour (%)	P-value
**All clients**	9838 (27.1%)	11030 (31.7%)	<0.001***	3357 (33.5%)	3704 (37.2%)	<0.001***
**Heterosexual females**	2662 (7.3%)	3217 (8.9%)	<0.001***	861 (8.6%)	1066 (10.7%)	<0.001***
**Heterosexual males**	3355 (9.3%)	3601 (9.9%)	<0.001***	1174 (11.7%)	1215 (12.2%)	0.98
**Female sex workers**	947 (2.6%)	796 (2.2%)	0.0055**	335 (3.3%)	262 (2.6%)	0.0031**
**Men who have sex with men**	2834 (7.8%)	3372 (9.3%)	<0.001***	972 (9.8%)	1147 (11.5%)	<0.001***
**Male sex workers**	169 (0.5%)	158 (0.4%)	0.82	59 (0.6%)	57 (0.6%)	0.88

*p<0.05;

**p<0.01;

***p<0.001.

A total of 2243 and 2589 STIs (defined in the Method section) were detected in the monthly and quarterly testing period, respectively (Table 3). There was a significant increase in prevalence of chlamydia (from 5.8% to 6.7%; *p*<0.001), gonorrhoea (from 3.1% to 4.3%; *p*<0.001) and trichomonas (from 0.2% to 0.5%; *p*<0.020) but a decrease in *M genitalium* (from 7.3% to 5.9%; *p* = 0.038) among all clients at MSHC during the period. The overall proportion of HIV (from 0.4% to 0.3%; *p* = 0.16) and syphilis (from 0.9% to 0.7%; *p* = 0.07) infections among all clients remained similar in the monthly and quarterly testing period. A two-fold increase in chlamydia (from 4.4% to 8.2%; *p* = 0.028) and gonorrhoea (from 5.1% to 10.1%; *p* = 0.011) was observed in male sex workers but no changes in other STIs. An increase in gonorrhoea cases as proportion of those tested was seen in MSM (from 6.1% to 7.1%; *p* = 0.014). There was an increase in trichomonas as the proportion of those tested in FSW (from 0.1% to 0.4%; *p* = 0.046); however, the proportion of individual FSW diagnosed with trichomonas in the two periods was not significantly different (from 0.4% to 0.6%, *p* = 0.61), and the proportion of individual FSW diagnosed with gonorrhoea infection in the two periods fell from 1.4% to 0.6% (*p* = 0.02). No significant changes in the other STI cases were observed in both heterosexual females and males during the period.

**Table 3:** Comparison of number of diagnosed STI cases among different categories of clients at the MSHC in the monthly and quarterly testing period.

STIs	Monthly/Quarterly testing period					
	All clients	Heterosexualfemales	Heterosexualmales∧	Female sexworkers&	Men who havesex with men	Male sexworkers
**Chlamydia**	1354 (5.8%)/1562 (6.7%) ***p*** **<0.001** ***	305 (6.7%)/393 (7.0%) *p* = 0.58	464 (7.7%)/496 (7.6%) *p* = 0.82	91 (1.5%)/64 (1.9%) *p* = 0.14	492 (7.4%)/599 (7.9%) *p* = 0.28	19 (4.4%)/28 (8.2%) ***p*** ** = 0.028** *
**Gonorrhoea**	504 (3.1%)/635 (4.3%) ***p*** **<0.001** ***	13 (0.5%)/12 (0.4%) *p* = 0.47	55 (5.5%)/64 (6.7%) *p* = 0.25	22 (0.4%)/8 (0.2%) *p* = 0.33	410 (6.1%)/548 (7.1%) ***p*** ** = 0.014** *	21 (5.1%)/31 (10.1%) ***p*** ** = 0.011** *
***M genitalium***	173 (7.3%)/197 (5.9%) ***p*** ** = 0.038** *	30 (6.1%)/39 (5.8%) *p* = 0.87	78 (6.1%)/85 (6.0%) *p* = 0.98	5 (3.6%)/13 (8.7%) *p* # = 0.091	60 (6.6%)/60 (5.4%) *p* = 0.25	5 (8.9%)/3 (6.1%) *p* # = 0.72
**Syphilis**	135 (0.9%)/122 (0.7%) *p* = 0.074	0 (0%)/0 (0%) *p* = N/A	9 (0.2%)/10 (0.3%) *p* = 0.97	4 (0.1%)/0 (0%) *p* # = 0.061	120 (1.9%)/112 (1.5%) *p* = 0.081	8 (2.1%)/3 (0.9%) *p* # = 0.24
**Trichomonas**	13 (0.2%)/16 (0.5%) ***p*** ** = 0.020** *	5 (0.2%)/11 (0.5%) *p* = 0.10	0 (0%)/0 (0%) *p* = N/A	8 (0.1%)/5 (0.4%) ***p*** # ** = 0.046** *	0 (0%)/0 (0%) *p* = N/A	0 (0%)/0 (0%) *p* # = N/A
**HIV**	64 (0.4%)/57 (0.3%) *p* = 0.16	0 (0%)/0 (0%) *p* = N/A	3 (0.1%)/1 (0.03%) *p* # = 0.35	0 (0%)/0 (0%) *p* = N/A	60 (1.0%)/55 (0.8%) *p* = 0.13	3 (0.9%)/0 (0%) *p* = 0.25
**Total**	2243/2589	353/455	609/656	130/90	1142/1374	56/65

Note: The numbers represent the number of positive cases detected, and the bracketed percentages represent the prevalence of infection in the population (i.e. number of positive cases divided by number of laboratory tests ordered).

*p<0.05;

**p<0.01;

***p<0.001.

# Fisher’s exact test.

∧Heterosexual men (males) are only tested for gonorrhoea when they have symptoms because gonorrhoea is rare in heterosexual men. Only 936 heterosexual men were tested for gonorrhoea in the monthly and 875 tested in the quarterly period.

& There was large number of repeated swab tests performed (i.e. chlamydia, gonorrhoea, *M genitalium* and trichomonas) in FSW due to the sex work regulation. The prevalence of FSW was adjusted to number of infected FSW individuals divided by the number of FSW individuals tested at least once in the period. The adjusted prevalence of chlamydia in FSW was: 81 (5.1%)/59 (4.0%) [*p* = 0.16]; gonorrhoea: 22 (1.4%)/8 (0.6%) [*p* = 0.020]; *M genitalium*: 5 (4.5%)/9 (8.0%) [*p* = 0.28]; and trichomonas: 7 (0.4%)/5 (0.6%) [*p* = 0.61].

There was a significant increase in STIs detected per 100 h of consultation time among all clients (from 22.4 to 26.0 cases/100 h; *p*<0.001), as well as for every 100 clinical consultations (from 6.2 to 7.4 cases/100 consultations; *p*<0.001) (Table 4). There was a significant increase in STI detection rates among MSM (from 8.7 to 10.8 cases/100 consultations; *p*<0.001) and male sex workers (from 7.9 to 13.0 cases/100 consultations; *p* = 0.003) during the period. The STI detection rate per 100 clinical consultations in FSW increased from 2.1 to 2.6 cases/100 consultations (*p* = 0.065), as well as for every 100 h of consultation time (from 6.7 to 7.8 cases/100 h; *p* = 0.13) between the monthly and quarterly testing period; though such increases were not statistically significant. The STI detection rate in FSW (2.1–2.6 cases/100 consultations) remained substantially lower in comparison with the heterosexual male (6.4–6.7 cases/100 consultations) and female population (4.8–5.3 cases/100 consultations).

**Table 4:** Comparison of the detection rates for selected STI among different categories of clients at the MSHC in the monthly and quarterly testing period.

	STI rate/100 consultations (95% CI)			STI rate/100 h (95% CI)		
Clients risk group	Monthly testingperiod	Quarterly testingperiod	P-value	Monthly testingperiod	Quarterly testingperiod	P-value
**All clients**	6.2 (6.0, 6.4)	7.4 (7.2, 7.7)	<0.001***	22.4 (21.6, 23.2)	26.0 (25.1, 26.9)	<0.001***
**Heterosexual females**	4.8 (4.4, 5.4)	5.3 (4.9, 5.8)	0.09	17.5 (15.9, 19.2)	18.9 (17.4, 20.5)	0.14
**Heterosexual males**	6.4 (5.9, 6.9)	6.7 (6.2, 7.2)	0.23	24.3 (22.6, 26.0)	25.3 (23.7, 27.0)	0.23
**Female sex workers**	2.1 (1.8, 2.5)	2.6 (2.1, 3.2)	0.065	6.7 (5.7, 7.9)	7.8 (6.4, 9.5)	0.13
**Men who have sex** **with men**	8.7 (8.3, 9.2)	10.8 (10.3, 11.4)	<0.001***	32.7 (31.1, 34.2)	36.6 (35.1, 38.2)	0.002**
**Male sex workers**	7.9 (6.1, 10.1)	13.0 (10.3, 16.2)	0.003**	27.8 (22.1, 34.4)	43.1 (34.5, 49.8)	0.013*

*p<0.05;

**p<0.01;

***p<0.001.

We have estimated the total costs for testing FSW at MSHC was AU$672,000 and AU$426,000 in the monthly and quarterly testing period, respectively (Table 5). Thus, approximately AU$247,000 was diverted away from screening FSW towards other clients with the change from monthly to quarterly testing. This large cost savings was mainly due to a reduction in clinical consultations and the number of laboratory tests ordered for FSW.

**Table 5:** Comparison between the costs associated with female sex workers before and after the change of testing frequency.

Items	Unit costs (AU$)	Monthly testing period		Quarterly testing period		Sources [13]
		No. of units	Total (AU$)	No. of units	Total (AU$)	
Swab test for chlamydia	21.50	5,964	128,226.00	3,299	70,928.50	MBS Item 69316
Swab test for gonorrhoea and/or trichomonas	25.35	5,984	151,694.40	3,228	81,829.80	MBS Item 69312
Screening test for HIV and syphilis	36.00	2,928	105,408.00	2,973	107,028.00	MBS Item 69390
Screening test for syphilis	24.65	13	320.45	15	369.75	MBS Item 69387
Screening test for HIV	13.35	7	93.45	11	146.85	MBS Item 69384
Clinical consultation (Level B)	36.30	2,814	102,148.20	1,178	42,761.40	MBS Item 23
Clinical consultation (Level C)	70.30	2,616	183,904.80	1,729	121,548.70	MBS Item 36
TOTAL			671,795.30		426,613.00	
**COST SAVED**					**247,182.30**	

## Discussion

This study showed a significant reduction in clinical consultations in FSW (∼40%) following a reduction in the frequency of STI screening of sex workers in Victoria from monthly to quarterly. The additional clinical capacity that this change created was filled with more symptomatic individuals, who were more likely to have STI diagnosed. The less frequent screening did not result in significantly higher rates of STI per 100 consultations or hours in FSWs, and remained two to four fold lower than all clients seen in the clinic. Up to a quarter of a million dollars annually was diverted away from screening towards higher risk clients. There was a substantial benefit to the Victorian community with an additional 346 cases of STIs diagnosed with no additional resources. The overall benefit to Victoria of this change in frequency has been substantial and provides further evidence to inform effective public health policy for sex workers.

Several limitations in this study should be noted. First, our findings are based on one sexual health clinic and it is possible that the changes we observed did not occur in other clinical settings. Second, our findings are relevant to sex workers working under a licensing regime with low prevalence of STI, it may not be appropriate to other settings with high STI rates and low condom use rate among FSW. Third, our study can only analyse FSW who were tested and we cannot infer our observed changes were seen among all FSW working in Melbourne. Fourth, we believe the savings on FSW is an underestimate of all costs because they do not account for the time of the individual sex workers to attend consultations. Fifth, this is a retrospective study and the data obtained previously might not fit in the framework of this study. Incomplete data may occur in the use of secondary data. Sixth, our costs have been estimated assuming that all sex workers were seen by doctors only because this is the case everywhere other than MSHC and even at MSHC doctors see a large proportion of clients. We acknowledge that we may have overestimated the specific cost savings at MSHC because of this reason, but they allow generalisation of costs to Victoria as a whole.

There was another change at MSHC that may have influenced these results. In January 2013, we changed our policy to allow MSM to obtain their HIV test results by phone rather than by face-to-face consultation. This may have resulted in the non-significant (*p* = 0.20) reduction in clinical consultations in MSM in the quarterly testing period. It is also possible that the reduction in this denominator contributed to the higher proportion of MSM who were symptomatic or the rise in STI diagnoses per 100 consultations or hours.

Our findings not only reflect a significant impact at MSHC, but also over the entire state of Victoria. There is no data set that identifies sex workers who attend general practitioners so it is not possible to undertake the same analysis that we did at MSHC. Unfortunately, there is also no record of the number of sex workers working in Victoria although the local sex worker organisation estimates that about half of sex works may attend MSHC for screening (Skelsey G, personal communication, 2013). If all Victorian FSWs are assumed to be tested quarterly, the total health care costs saved will be about half a million dollars in addition to the benefit of making other clinical services more accessible. In addition, there is no noticeable change in STI detection rate in FSW when the screening interval is reduced from monthly to quarterly. The quarterly screening regimen could result in a delayed diagnosis of STI or may increase the duration of infection in FSWs but it is unlikely to result in significant transmission of STIs to their clients if condoms are used 100% of the time [9]. Indeed previous research has shown that non-paying private partners, with whom they do not use condoms, are the usual source of STIs in FSWs [5].

More important than the cost saving achieved by reducing the screening interval that provides no discernible benefit, is the public health benefit associated with improving the clinical services for those at high risk of STI. Accessible health services have been shown to be a critical component of effective STI control [14]. The reduction in STI screening in sex workers allowed more higher-risk individuals, such as MSM and the general community to access the sexual health services without additional staff costs. The data confirmed this and showed more time and consultations were spent on individuals with symptomatic conditions. We did not evaluate the impact on male sex workers because there are relatively few male sex workers in Victoria and the majority (84.9%; 1028/1211) have private male partners and have STI rates comparable to MSM in general [15]. Male sex workers are more a complicated population and at a higher-risk of HIV/STI than FSW [15], [16]. In addition, the majority licensed brothels in Victoria (∼98%) do not have male sex workers [17]; and hence, screening regimen on male sex workers is a challenge and should be addressed differently.

One potential benefit of the reduction in sex worker screening is that it may encourage sex workers who work in illegal brothels to move back into the legal system now that the onerous monthly screening program has been removed. Our centre saw a greater number of sex workers supporting this possibility. If this is so, it is an additional advantage of the change because it is believed that women working in the illegal industry have higher rates of STIs and use condoms less [18]. Occupational Health and Safety is also much greater for women working within the legal system.

To our knowledge, this is the first study to investigate the impact of the change in STI screening frequency for sex workers on a sexual health service. The findings may have important implications for other countries seeking to create the optimal legislation for sex workers in their countries. We have shown that quarterly STI screening of sex workers is cheaper and more effective than the monthly screening at a community level which is of considerable relevance to public health policy and legislatory reform for those working in the field.
